# RNAi-mediated knockdown of ERK1/2 inhibits cell proliferation and invasion and increases chemosensitivity to cisplatin in human osteosarcoma U2-OS cells *in vitro*

**DOI:** 10.3892/ijo.2011.1303

**Published:** 2011-12-15

**Authors:** HAIPENG SI, CHANGLIANG PENG, JINGJING LI, XIQIAN WANG, LIANWEN ZHAI, XIAOFENG LI, JIANMIN LI

**Affiliations:** 1 Department of Orthopaedics, Second Affiliated Hospital, Shandong University, 247 Beiyuan Da Street, Jinan 250033; 2 Key Laboratory of Cardiovascular Remodeling and Function Research, Shandong University School of Medicine, 44 Wen Hua Xi Road, Jinan 250012; 3 Department of Orthopaedics, Qilu Hospital, Shandong University, 107 Wen Hua Xi Road, Jinan 250012, P.R. China

**Keywords:** ERK1/2, osteosarcoma, siRNA, chemosensitivity

## Abstract

Osteosarcoma is the most common primary malignancy of the bone. There have been some advances in surgical and chemotherapeutic strategies, but it is still a tumor with a high mortality rate in children and young adults. Mitogen-activated protein kinase/extracellular signal regulated kinase (ERK) pathway plays an essential role in the development and progression of various tumors. ERK1/2 is a key component of this pathway and hyperactivated in different tumors including osteosarcoma. This study aimed to investigate whether downregulation of ERK1/2 by siRNA (small interfering RNA) could inhibit cell proliferation and invasion and increase chemosensitivity to cisplatin in human osteosarcoma U2-OS cells *in vitro*. Results showed that the downregulation of ERK1/2 expression by siRNA in human osteosarcoma cells significantly inhibited cell proliferation and invasion *in vitro*. Furthermore, ERK1/2 knockdown led to cell arrest in the G1/G0 phase of the cell cycle, and eventual apoptosis and chemosensitivity enhancement in tumor cells. Our data reveal that RNAi-mediated downregulation of ERK1/2 expression can lead to potent antitumor activity and chemosensitizing effects in human osteosarcoma.

## Introduction

Osteosarcoma (OS) remains the most common primary malignant bone cancer affecting children and adolescents (1). Although the combination of modern surgery and systemic chemotherapy has improved OS treatment dramatically, no substantial change in survival has been seen over the past 20 years (2). For this reason, understanding the mechanisms underlying OS as well as identifications of new molecular targets are of great importance.

Mitogen-activated protein kinases (MAPK) are a family of serine/threonine protein kinases widely conserved among eukaryotes and plays essential roles in many cellular programs such as cell proliferation, cell differentiation, cell movement and cell death. The classical MAPK pathway consists of RAS, RAF, MEK and ERK, sequentially relaying proliferative signals generated at the cell surface receptors and through cytoplasmic signaling into the nucleus (3). Under normal cellular conditions, this pathway is stimulated by the binding of mitogens, hormones, or neurotransmitters to receptor tyrosine kinases, which upon dimerization triggers the activation of oncogenic RAS to increase cellular RAS-GTP levels. Activated RAS then triggers the formation of the ‘MAPK complex’ with downstream RAF, MEK1/2, ERK1/2. Once activated, ERK1/2 regulates the expression of several genes involved in cell proliferation, differentiation and survival by phosphorylating nuclear transcription factors such as ETS, ELK-1, MYC or indirectly by targeting intracellular signaling molecules such as p90-RSK. The ERK1/2 also has profound effects on the regulation of apoptosis by the post-translational phosphorylation of apoptotic regulatory molecules including Bad, Bim, Mcl-1, caspase 9 and Bcl-2 (3–5). Constitutive ERK1/2 activation is often due to overexpression or mutation of upstream receptor tyrosine kinases such as EGFR, PDGFR, or VEGFR, increased expression of growth factor ligands, or mutational activation of Ras and its downstream effectors (6).

The involvement of constitutive ERK1/2 activation in the proliferation and tumoral progression has been largely reported in many cancer cells including malignant melanomas (7), human hepatocellular carcinoma (8), esophagogastric cancer (9), breast cancer (10), renal cell carcinomas (11) and leukemia (12). The hyperactivation of ERK1/2 also has been shown to promote resistance to chemotherapy drugs in many cancer cells (4,5,13). Therefore, ERK1/2 could be a good therapeutic target, as agents inhibiting the action of ERK1/2 might prevent tumor cell proliferation, promote apoptosis and reverse resistance to therapy.

Although ERK1/2 was shown to be overexpressed in OS cells and has been implicated as playing important roles in the development of OS (14,15), the exact function of ERK1/2 in OS growth and progression is not fully understood. Inhibition of ERK1/2 has been shown to enhance the chemosensitivity of the cancer cells towards the anticancer drugs (4,5). But the relationship between ERK1/2 expression and response to chemotherapy in human osteosarcoma cells has not been elucidated. Small interfering RNA (siRNA) is a highly specific and efficient tool to silence target genes (16). In the present study, we employed siRNA targeting ERK1/2 to explore the potential of new therapeutic targets in the treatment of OS. Our results suggest that knockdown of ERK1/2 could inhibit OS proliferation and invasion, and could significantly increase the chemosensitivity to cisplatin-induced apoptosis in OS cells. Thus, ERK would be a good molecular target for OS therapy.

## Materials and methods

### Cell culture and siRNA transfection

Human osteosarcoma cell line (U2-OS) was obtained from the American Type Culture Collection (ATCC, Rockville, MD, USA). Cells were cultured in Dulbecco’s modified Eagle’s medium (Gibco RL, Grand Island, NY, USA) supplemented with 10% fetal bovine serum, penicillin (100 U/ml) and streptomycin (100 μg/ml) in a humidified 5% CO_2_ atmosphere. The human ERK1 and ERK2 specific siRNA were based on NCBI Reference Sequences (GenBank: ERK1: NM_002746.2 and ERK2: NM_002745.4). ERK1/2 siRNA and scrambled control siRNA (siCONTROL) were purchased from Cell Signaling Technology (Beverly, MA, USA). All of the siRNA transfections were performed using Lipofectamine 2000 (Invitrogen, Carlsbad, CA, USA) in Opti-MEM (Invitrogen) according to the manufacturer’s protocol with a final siRNA concentration of 100 nM. The transfection reagent was removed after 12 h and the cells were harvested after 48 h.

### Real-time PCR analysis

Total RNA was isolated from transfected cells using TRIzol reagent (Invitrogen). RNA was first retro-transcribed using TaqMan^®^ Reverse Transcription Kit (Applied Biosystems, Foster City, CA, USA) and then real-time PCR was carried out using and TaqMan SYBR Green Master Mix (Applied Biosystems). β-actin was applied as an internal control. The following primers were used: ERK1: 5′-CGCTACACGC AGTTGCAGTACA-3′ forward and 5′-AAGCGCAGCAGG ATCTGGA-3′ reverse; ERK2: 5′-TGTTCCCAAATGCTG ACTCCAA-3′ forward and 5′-TCGGGTCGTAATACTGC TCCAGATA-3′ reverse; β-actin: 5′-GGCGGCACCACCATG TACCCT-3′ forward and 5′-AGGGGCCGGACTCGTCATA CT-3′ reverse. The comparative Ct method was used to calculatethe relative abundance of mRNA compared with that of β-actin expression (17).

### Western blot analysis

U2-OS cells were lysed in boiling buffer (10 mM) containing 1% SDS and boiled for 5 min. After centrifugation at 20,800 × g for 5 min at 4°C, the supernatant was collected and protein concentration was measured using the BCA assay (Pierce, Rockford, IL, USA). A total of 30 μg of protein per sample was resolved by 10% SDS-PAGE, followed by electro-transfer to PVDF membranes (Millipore, Bedford, MA, USA). After transfer, the membranes were blocked with 5% skim milk and probed with specific primary antibodies overnight followed by 1 μg/ml horseradish peroxidase conjugated secondary antibody. Antibodies against p44/42 MAPK (Erk1/2) antibody (1:1000 dilution) and phospho-p44/42 MAPK (Erk1/2) (Thr202/Tyr204) antibody (1:1000 dilution) were from Cell Signaling Technology (Beverly, MA, USA); antibodies for Bax (1:500 dilution), Bcl-2 (1:500 dilution), Mcl-1 (1:500 dilution), β-actin (1:500 dilution) and anti-mouse and anti-rabbit antibodies linked with horseradish peroxidase were from Santa Cruz (Santa Cruz, CA, USA). Western blotting was performed as described previously (18).

### MTT assays for cell proliferation

Briefly, 3×10^3^ cells per well were plated in a 96-well plate. After 48 h of incubation, cells were treated with ERK1/2 siRNA, scrambled control siRNA or with medium alone. At various time-points, 3-(4,5-dimethyl-thiazol-2-yl)-2, 5-diphenyltetrazolium bromide (MTT) (Sigma, St. Louis, MO, USA) was added into each well at a final concentration of 5 mg/ml and allowed to incubate at 37°C for 4 h. DMSO (150 μl) was then added to stop the reaction and measured with an ELISA reader (Bio-Rad, Hercules, CA, USA) at a wavelength of 570 nm. Viability of cells was expressed relative to theoretical absorbance (A). Each experiment was performed in triplicate.

### Cell cycle analysis by flow cytometer

U2-OS cells (5×10^5^) were seeded into each well of a 6-well culture plate and incubated overnight. Cells were then transfected with the indicated siRNAs. After an additional incubation for 48 h, cells were detached and fixed with 500 μl of 70% ethanol at −20°C for 2 h. Subsequently, the cells were washed twice with PBS then stained with propidium iodide (50 μg/ml propidium iodide and 100 μg/ml RNase A in PBS) at 37°C for 30 min. Cell cycle analysis was performed on a FACScan flow cytometer (Becton-Dickinson, San Jose, CA, USA).

### Annexin V-propidium iodide (AnnV/PI) staining apoptosis test

After siRNA transfection and cisplatin (10 μg/ml) (Sigma) treatment, cells in each well were harvested and cell apoptosis was detected by Annexin V-FITC/PI staining method. The experiments were done in triplicate for each sample, and analyses were performed using a FACScan flow cytometer (Becton-Dickinson) in accordance with the manufacturer’s guidelines.

### Invasion assay

Cell invasion assay was performed in BioCoat transwell chambers (Corning Costar, Cambridge, MA, USA) with uncoated porous inserts (pore size 8 μm) as previously described (19). Matrigel™ Matrix (BD Biosciences, San Jose, CA, USA) was diluted 1:5 using serum-free DMEM medium and added to the transwell inserts. Cells at 1×10^4^/ml from different groups were spread on the transwell inserts pre-coated with Matrigel in 24-well plates and cultured in serum-free DMEM. Medium containing 10% FBS was added to the lower chamber as a chemoattractant. After 24-h incubation at 37°C in a 5% CO_2_ incubator, the insert was washed with PBS, and cells on the upper surface of the insert were wiped off using a cotton swab. Cells that migrated to the bottom surface of the insert were fixed with methanol and stained by Giemsa. The number of cells was counted under the microscope from randomly selected five fields (magnification ×400).

### Statistical analysis

Results are expressed as means ± standard deviation (SD). Statistical analyses were performed using SPSS statistical software (SPSS Inc., Chicago, IL, USA). The statistical significance of the results was determined using the Student’s t-test. Data were considered significant at P<0.05.

## Results

### siRNA targeting ERK1/2 knocks down ERK1/2 expression

**Figure 1:**
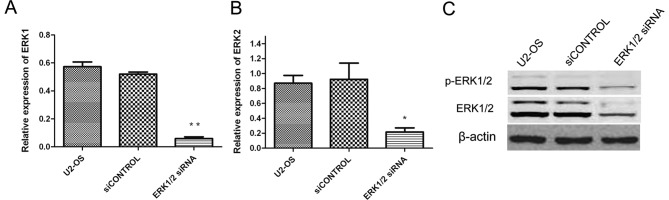
Specific downregulation of ERK1/2 mRNA and protein expression by ERK1/2 siRNA. Cells were transfected with 100 nM ERK1/2 siRNA or scramble siRNA (siCONTROL) for 48 h, and the relative mRNA levels of ERK1 and ERK2 were quantified by real-time PCR analysis (A and B) and the relative protein levels of ERK1/2 and phosphorylated ERK1 and ERK2 (pERK1/2) were determined by Western blotting (C). Data were normalized by using β-actin as an internal standard. ^*^P<0.05 and ^**^P<0.01 vs. siCONTROL.

Since ERK1/2 levels are significantly higher in tumor cells (including osteosarcoma) than that in normal cells, we sought to determine whether the synthetic ERK1/2 siRNA could inhibit the expression of ERK1/2 gene in U2-OS cells. Forty-eight hours after siRNA transfection, ERK1/2 mRNA and protein were measured by quantitative real-time PCR and Western blotting, respectively. As shown in Fig. 1, ERK1/2 siRNA could specifically and efficiently suppress ERK1/2 expression at both mRNA and protein levels compared to cells treated with siCONTROL and the untreated cells.

### ERK1/2 knockdown inhibits osteosarcoma cell growth

**Figure 2:**
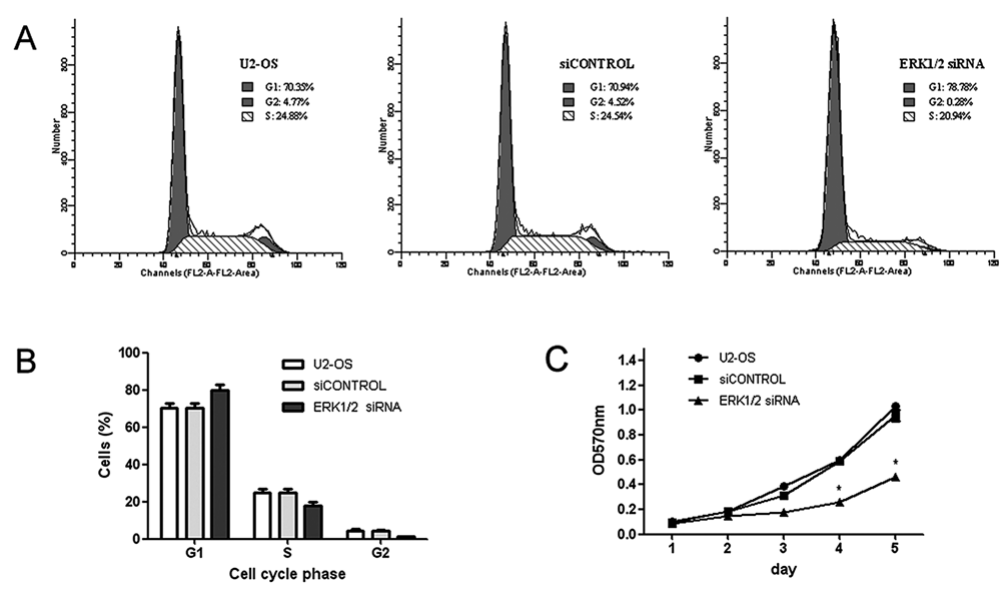
RNAi-mediated downregulation of ERK1/2 reduces U2-OS cell proliferation. (A) Cell cycle analysis of U2-OS was carried out by flow cytometry after 48-h transfection of ERK1/2 siRNA or siCONTROL. (B) Cell proliferation was assessed at the indicated times by MTT assays. Data are from three independent experiments. ^*^P<0.05, ^**^P<0.01 compared to the control group.

To determine whether ERK1/2 siRNA had an inhibitory effect on U2-OS cell growth, we first performed determination of cell proliferation with MTT assay. Fig. 2C showed that the growth curves for ERK1/2 knockdown cells were significantly lower than those for control cells for 5 days of incubation. Furthermore, the cell cycle distribution of control and transfected cells was analyzed by flow cytometry. As shown in Fig. 2A, there was a significant increase in the percentage of cells in G1 phase after transfection with ERK1/2 siRNA. In detail, the percentage of cells at G1 phase increased by 7.84% in the U2-OS cells transfected with the ERK1/2 siRNA while the percentage of cells at S phase decreased by 3.60% (Fig. 2A). The cells transfected with the siCONTROL were set as the control group. This result indicated that siRNA arrested the cells in G0 and G1 phases, delayed the progression of cell cycle and inhibited cell proliferation.

### Downregulation of ERK1/2 inhibits osteosarcoma cell invasion

**Figure 3:**
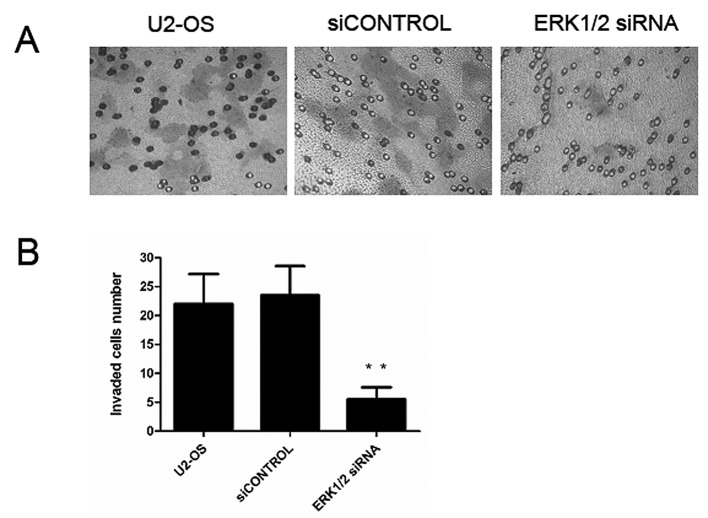
Downregulation of ERK1/2 suppresses osteosarcoma cell migration. (A) U2-OS, siCONTROL and ERK1/2 siRNA cells were induced to invade through Matrigel-coated transwell membranes. After 48 h, cells were fixed, stained and counted. (B) The cell numbers of ERK1/2 siRNA treated groups were significantly less than U2-OS and siCONTROL groups (P<0.01).

Cancer cell migration and invasion play very important roles in cancer metastasis. So, we further studied the effect of ERK1/2 suppression on the invasion in U2-OS cells. The results showed that the migratory ability was significantly reduced through Matrigel coated chamber membranes (P<0.01, Fig. 3) compared with the untreated group, or the siCONTROL group. These results indicate that silencing of ERK1/2 plays an inhibitory effect on the invasion of U2-OS cells.

### Inhibition of ERK1/2 increases sensitivity of U2-OS cells to chemotherapy

**Figure 4:**
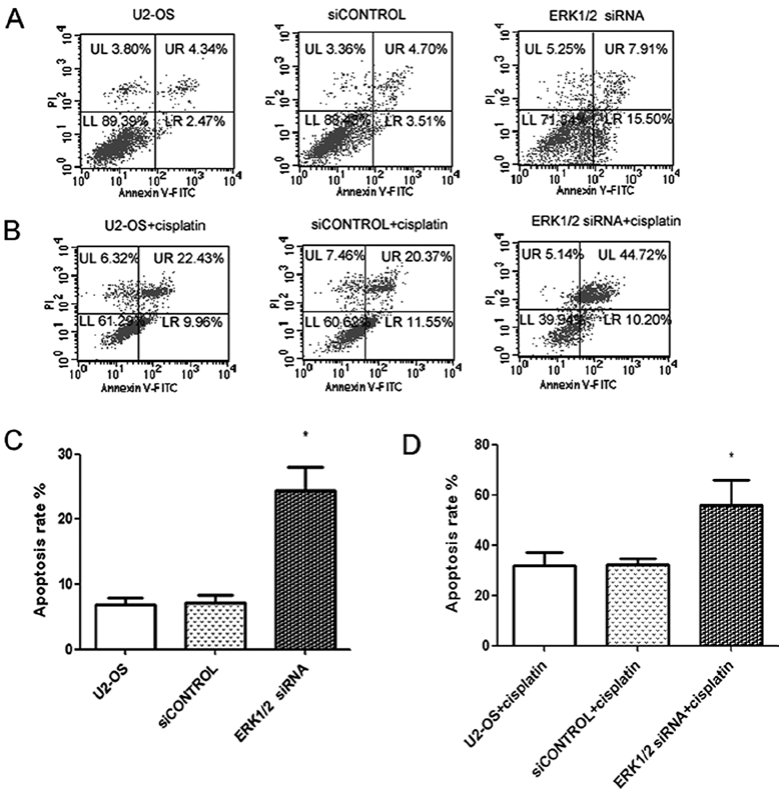
ERK1/2 depletion induces apoptosis and enhances chemosensitivity. (A and C) ERK1/2 inhibition by siRNA caused apoptosis in U2-OS cells. (B and D) ERK1/2 knockdown sensitized U2-OS cells to cisplatin treatment. After 48-h treatment with indicated siRNAs, cells were treated with 10 μg/ml cisplatin for 24 h, and then cells were double stained with annexin V-FITC and PI followed by FACS analysis. FACS analysis scatter-grams of Annexin V/PI staining displays four different cell populations marked as: double negative (unstained) cells showing live cell population (lower left), Annexin V positive and PI negative stained cells showing early apoptosis (lower right), Annexin V/PI double-stained cells showing late apoptosis (upper right), and finally PI positive and Annexin V negative stained cells showing dead cells (upper left). ^**^P<0.01 vs siCONTROL.

Upregulation of the ERK1/2 gene has been reported to be closely related to chemotherapy resistance, and inhibition of the ERK1/2 gene has been shown to increase the sensitivity of tumor cells to chemotherapeutic agents (20,21). These exciting data prompted us to investigate the possibility of the combination of cisplatin treatment and ERK1/2 depletion as a clinical strategy for osteosarcoma chemotherapy. The effect of ERK1/2 siRNA and cisplatin on cell apoptosis was examined in U2-OS cell line by flow cytometric analysis. As shown in Fig. 4A and C, cell apoptosis was induced after transfection with ERK1/2 siRNA at 48 h. However, cell apoptosis was further enhanced when cells were treated with 10 μg/ml cisplatin (Fig. 4B and D), suggesting that combining ERK1/2 inhibition with cisplatin increased the incidence of apoptosis.

### ERK1/2 knockdown decreases the expression of anti-apoptotic proteins Bcl-2 and Mcl-1 while increases the expression of proapoptotic protein Bax in U2-OS cells

**Figure 5:**
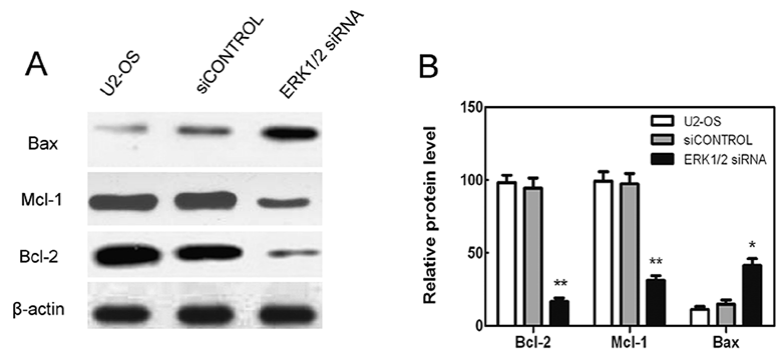
Inhibition of ERK1/2 decreases expression of Bcl-2 and Mcl-1, whereas it induces Bax expression in U2-OS cells. The cells were treated with indicated siRNAs for 48 h, and 30 μg cell lysate proteins were separated on 10% sodium dodecyl sulfate-polyacrylamide gel electrophoresis and detected using specific antibodies. Immunoblotting of actin was shown to demonstrate loading equivalency. Inhibition of ERK1/2 significantly reduced expression of Bcl-2 and Mcl-1, whereas it induced Bax expression in U2-OS cells (A). The columns represent the ratio of Bcl-2, Mcl-1 and Bax to actin (B). Data represent the mean ± SD of three independent experiments. ^*^P<0.05 and ^**^P<0.01 vs. siCONTROL.

In order to gain a better understanding of the mechanism leading to cell death, the tumor cells were analyzed for the alteration of different kinds of anti-apoptotic and pro-apoptotic Bcl-2 family proteins. While ERK1/2 knockdown caused downregulation of Bcl-2 and Mcl-1, increased levels of Bax were evident (Fig. 5). Levels of Bcl-2, Mcl-1 and Bax did not show a clear change as the cell was transfected with the control siRNA (Fig. 5). Therefore, ERK1/2 knockdown caused the increased cell death by activating pro-apoptotic Bcl-2 members, while inhibiting the anti-apoptotic ones.

## Discussion

Osteosarcoma (OS) is the most frequent primary bone sarcoma, accounting for approximately 20% of all primary sarcomas in bone and approximately 5% of pediatric tumors overall (1). OS is characterized by aggressive invasion, early metastasis and resistance to existing chemotherapeutic agents or radiotherapy. Despite advances in surgical and chemotherapeutic strategies, the prognosis of osteosarcoma patients remains unfavorable (22). The failure to improve outcomes with treatment intensification indicates the need for novel and effective antitumor strategies, such as targeted therapy.

Constitutive activation of ERK1/2 has been implicated in a wide variety of processes in the cancer cell during the regulation of cell mortality, apoptosis, angiogenesis, invasion and metastasis, and cell division cycle (4,23). On the basis of the importance of ERK1/2 in tumor progression and survival, ERK1/2 recently has been considered as a molecular target for cancer chemotherapeutics (23–25). Studies with genetic approaches including antisense oligonucleotides and RNA interference (RNAi) have demonstrated that inhibition of ERK1/2 signaling suppresses tumor growth and induces apoptosis *in vitro* and *in vivo* (26–28). Moreover, pharmacological agents including gefitinib and sorafenib have also been demonstrated to be potential anticancer drugs due to their inhibitory effects on ERK1/2 signaling (29,30).

Inhibiting specific gene expression by RNAi has become an important method of cancer treatment (31,32). Recently, ERK1/2 signal pathway was shown to be overexpressed in OS cells and has been proposed as a novel marker of poor outcome in OS (14,33,34). To explore whether ERK1/2 could become a potential molecular target for gene therapy of osteosarcoma, we employed RNAi technology to downregulate ERK1/2 expression in a human cell line of osteosarcoma, U2-OS. Comparing with control group, the mRNA and protein expressions were decreased significantly in the cells transfected with the ERK1/2 siRNA. These results indicated that ERK1/2 siRNA could silence the expression of ERK1/2 effectively and specifically in U2-OS cells.

We next examined the consequences of U2-OS cells transfected with ERK1/2 siRNA. The proliferation rate was analyzed by MTT and the cell cycle distribution was tested by flow cytometry, then we found that ERK1/2 knocked down by siRNA suppressed cell proliferation and induced an accumulation in G1 phase concomitant with a decrease in the S phase in U2-OS cells *in vitro*. The results suggested that the observed growth-inhibitory effect of ERK1/2 siRNA on U2-OS cells could be mediated through modulation of cell cycle progression. Our results are in line with others who showed that inhibition of ERK1/2 signaling induced cell cycle arrest in G0/G1 phase and reduced proliferation in cancer cells (35,36). These results manifested that ERK1/2 plays an important role both in cell proliferation and cell cycle.

It is known that ERK1/2 phosphorylation and the subsequent activation of myosin light chain kinase can modulate focal contact dynamics in motile cells, promote migration and invasion (5,7). We further studied the effect of ERK1/2 suppression on the migration of U2-OS cells by mobility assays. The results showed that the migratory ability was significantly reduced through Matrigel coated chamber membranes compared with the control group. Therefore, there is a strong relationship between ERK1/2 and the invasion or migration ability of human osteosarcoma cells. These results are consistent with other recent reports that showed that inhibition of ERK1/2 signaling could reduce the migration and invasion of tumor cells (37,38).

An important downstream effect of ERK1/2 activation is the ERK1/2-dependent regulation of antiapoptotic Bcl-2 family genes (6). Bcl-2 and its dominant inhibitor Bax are key regulators of cell growth and apoptosis. The anti-apoptotic Bcl-2 proteins protect cells from apoptosis, while Bax accelerates cell death in response to certain apoptotic stimuli. Treatment with ERK1/2 siRNA induced apoptosis (Fig. 4A and C) with corresponding decrease in ERK1/2 activation, accompanied by the reduction of Bcl-2 and Mcl-1 and upregulation of the levels of Bax protein in U2-OS cells (Fig. 5). Such effects may be the important mechanisms of action of ERK1/2 induced apoptosis and suppressed the growth of the U2-OS cells. The displayed results correspond with other recent reports that showed that inhibition of ERK1/2 signaling was accompanied by growth inhibition and induction of apoptosis (39,40). But understanding the detailed molecular mechanisms needs further investigation.

Drug resistance is an important cause of treatment failure and mortality in OS patients. Cisplatin is one of the most commonly used anticancer drugs, but its application has been limited by the presence of cellular resistance (41). Upregulation of ERK1/2 was reported in multidrug resistant cancers cells (20). Numerous reports have shown convincing data that the inhibition of ERK1/2 could sensitize tumor cells to cisplatin-induced cell death (42–44). However, the relationship between ERK1/2 expression and response to chemotherapy in human osteosarcoma cells has not been disclosed. Here, we postulate that inhibition of ERK1/2 expression via RNAi could increase the chemosensitivity to cisplatin. As expected, knockdown of ERK1/2 expression contributed to sensitizing osteosarcoma cells to the anticancer drug cisplatin by increasing apoptosis.

In this report, we have shown that downexpression of ERK1/2 by siRNA inhibited the growth and invasion of U2-OS cells, and found that the knockdown of ERK1/2 made cancer cells more sensitive to cisplatin treatment and that the depletion of ERK1/2 enhanced cisplatin-induced apoptosis. Furthermore, we demonstrated that the knockdown of ERK1/2 regulates the expression level of Bcl-2, Mcl-1 and Bax. All these findings suggest that ERK1/2 may have the wide therapeutic and/or adjuvant application in the treatment of human osteosarcoma.

## References

[1] Mirabello L, Troisi RJ, Savage SA (2009). Osteosarcoma incidence and survival rates from 1973 to 2004: data from the Surveillance, Epidemiology, and End Results Program. Cancer.

[2] Akiyama T, Dass CR, Choong PF (2008). Novel therapeutic strategy for osteosarcoma targeting osteoclast differentiation, bone-resorbing activity, and apoptosis pathway. Mol Cancer Ther.

[3] Inamdar GS, Madhunapantula SV, Robertson GP (2010). Targeting the MAPK pathway in melanoma: why some approaches succeed and other fail. Biochem Pharmacol.

[4] McCubrey JA, Steelman LS, Abrams SL (2006). Roles of the RAF/MEK/ERK and PI3K/PTEN/AKT pathways in malignant transformation and drug resistance. Adv Enzyme Regul.

[5] McCubrey JA, Steelman LS, Chappell WH (2007). Roles of the Raf/MEK/ERK pathway in cell growth, malignant transformation and drug resistance. Biochim Biophys Acta.

[6] Eisenmann KM, van Brocklin MW, Staffend NA, Kitchen SM, Koo HM (2003). Mitogen-activated protein kinase pathway-dependent tumor-specific survival signaling in melanoma cells through inactivation of the proapoptotic protein bad. Cancer Res.

[7] Satyamoorthy K, Li G, Gerrero MR (2003). Constitutive mitogen-activated protein kinase activation in melanoma is mediated by both BRAF mutations and autocrine growth factor stimulation. Cancer Res.

[8] Chen L, Shi Y, Jiang CY, Wei LX, Wang YL, Dai GH (2011). Expression and prognostic role of pan-Ras, Raf-1, pMEK1 and pERK1/2 in patients with hepatocellular carcinoma. Eur J Surg Oncol.

[9] Barry OP, Mullan B, Sheehan D (2001). Constitutive ERK1/2 activation in esophagogastric rib bone marrow micrometastatic cells is MEK-independent. J Biol Chem.

[10] Sivaraman VS, Wang H, Nuovo GJ, Malbon CC (1997). Hyperexpression of mitogen-activated protein kinase in human breast cancer. J Clin Invest.

[11] Oka H, Chatani Y, Hoshino R (1995). Constitutive activation of mitogen-activated protein (MAP) kinases in human renal cell carcinoma. Cancer Res.

[12] Gregorj C, Ricciardi MR, Petrucci MT (2007). ERK1/2 phosphorylation is an independent predictor of complete remission in newly diagnosed adult acute lymphoblastic leukemia. Blood.

[13] Sridhar SS, Hedley D, Siu LL (2005). Raf kinase as a target for anticancer therapeutics. Mol Cancer Ther.

[14] Yang R, Piperdi S, Gorlick R (2008). Activation of the RAF/mitogen-activated protein/extracellular signal-regulated kinase kinase/extracellular signal-regulated kinase pathway mediates apoptosis induced by chelerythrine in osteosarcoma. Clin Cancer Res.

[15] Shimo T, Matsumura S, Ibaragi S (2007). Specific inhibitor of MEK-mediated cross-talk between ERK and p38 MAPK during differentiation of human osteosarcoma cells. J Cell Commun Signal.

[16] Bass BL (2000). Double-stranded RNA as a template for gene silencing. Cell.

[17] Schmittgen TD, Livak KJ (2008). Analyzing real-time PCR data by the comparative C(T) method. Nat Protoc.

[18] Iwagaki A, Choe N, Li Y, Hemenway DR, Kagan E (2003). Asbestos inhalation induces tyrosine nitration associated with extracellular signal-regulated kinase 1/2 activation in the rat lung. Am J Respir Cell Mol Biol.

[19] Van Golen KL, Wu ZF, Qiao XT, Bao LW, Merajver SD (2000). RhoC GTPase, a novel transforming oncogene for human mammary epithelial cells that partially recapitulates the inflammatory breast cancer phenotype. Cancer Res.

[20] Lin JC, Chang SY, Hsieh DS, Lee CF, Yu DS (2005). Modulation of mitogen-activated protein kinase cascades by differentiation-1 protein: acquired drug resistance of hormone independent prostate cancer cells. J Urol.

[21] Zhang D, LaFortune TA, Krishnamurthy S (2009). Epidermal growth factor receptor tyrosine kinase inhibitor reverses mesenchymal to epithelial phenotype and inhibits metastasis in inflammatory breast cancer. Clin Cancer Res.

[22] Bacci G, Longhi A, Versari M, Mercuri M, Briccoli A, Picci P (2006). Prognostic factors for osteosarcoma of the extremity treated with neoadjuvant chemotherapy: 15-year experience in 789 patients treated at a single institution. Cancer.

[23] Roberts PJ, Der CJ (2007). Targeting the Raf-MEK-ERK mitogen-activated protein kinase cascade for the treatment of cancer. Oncogene.

[24] Montagut C, Settleman J (2009). Targeting the RAF-MEK-ERK pathway in cancer therapy. Cancer Lett.

[25] Friday BB, Adjei AA (2008). Advances in targeting the Ras/Raf/MEK/Erk mitogen-activated protein kinase cascade with MEK inhibitors for cancer therapy. Clin Cancer Res.

[26] Gailhouste L, Ezan F, Bessard A (2010). RNAi-mediated MEK1 knock-down prevents ERK1/2 activation and abolishes human hepatocarcinoma growth in vitro and in vivo. Int J Cancer.

[27] Zeng P, Wagoner HA, Pescovitz OH, Steinmetz R (2005). RNA interference (RNAi) for extracellular signal-regulated kinase 1 (ERK1) alone is sufficient to suppress cell viability in ovarian cancer cells. Cancer Biol Ther.

[28] Koul S, Huang M, Chaturvedi L, Meacham RB, Koul HK (2004). p42/p44 mitogen-activated protein kinase signal transduction pathway regulates interleukin-6 expression in PC3 cells, a line of hormone-refractory prostate cancer cells. Ann NY Acad Sci.

[29] Liu L, Cao Y, Chen C (2006). Sorafenib blocks the RAF/MEK/ERK pathway, inhibits tumor angiogenesis, and induces tumor cell apoptosis in hepatocellular carcinoma model PLC/PRF/5. Cancer Res.

[30] Janmaat ML, Rodriguez JA, Gallegos-Ruiz M, Kruyt FA, Giaccone G (2006). Enhanced cytotoxicity induced by gefitinib and specific inhibitors of the Ras or phosphatidyl inositol-3 kinase pathways in non-small cell lung cancer cells. Int J Cancer.

[31] Lapteva N, Yang AG, Sanders DE, Strube RW, Chen SY (2005). CXCR4 knockdown by small interfering RNA abrogates breast tumor growth in vivo. Cancer Gene Ther.

[32] Susa M, Iyer AK, Ryu K (2010). Inhibition of ABCB1 (MDR1) expression by an siRNA nanoparticulate delivery system to overcome drug resistance in osteosarcoma. PLoS One.

[33] Pignochino Y, Grignani G, Cavalloni G (2009). Sorafenib blocks tumour growth, angiogenesis and metastatic potential in preclinical models of osteosarcoma through a mechanism potentially involving the inhibition of ERK1/2, MCL-1 and ezrin pathways. Mol Cancer.

[34] Do SI, Jung WW, Kim HS, Park YK (2009). The expression of epidermal growth factor receptor and its downstream signaling molecules in osteosarcoma. Int J Oncol.

[35] Gysin S, Lee SH, Dean NM, McMahon M (2005). Pharmacologic inhibition of RAF-->MEK-->ERK signaling elicits pancreatic cancer cell cycle arrest through induced expression of p27^Kip1^. Cancer Res.

[36] Sutter AP, Maaser K, Gerst B, Krahn A, Zeitz M, Scherubl H (2004). Enhancement of peripheral benzodiazepine receptor ligand-induced apoptosis and cell cycle arrest of esophageal cancer cells by simultaneous inhibition of MAPK/ERK kinase. Biochem Pharmacol.

[37] Shukla A, Hillegass JM, Macpherson MB (2011). ERK2 is essential for the growth of human epithelioid malignant mesotheliomas. Int J Cancer.

[38] Chen H, Zhu G, Li Y (2009). Extracellular signal-regulated kinase signaling pathway regulates breast cancer cell migration by maintaining slug expression. Cancer Res.

[39] Pellicano F, Simara P, Sinclair A (2011). The MEK inhibitor PD184352 enhances BMS-214662-induced apoptosis in CD34^+^ CML stem/progenitor cells. Leukemia.

[40] Trisciuoglio D, Iervolino A, Zupi G, Del Bufalo D (2005). Involvement of PI3K and MAPK signaling in bcl-2-induced vascular endothelial growth factor expression in melanoma cells. Mol Biol Cell.

[41] Zamble DB, Lippard SJ (1995). Cisplatin and DNA repair in cancer chemotherapy. Trends Biochem Sci.

[42] Mandic A, Viktorsson K, Heiden T, Hansson J, Shoshan MC (2001). The MEK1 inhibitor PD98059 sensitizes C8161 melanoma cells to cisplatin-induced apoptosis. Melanoma Res.

[43] Lu Y, Cederbaum A (2007). The mode of cisplatin-induced cell death in CYP2E1-overexpressing HepG2 cells: modulation by ERK, ROS, glutathione, and thioredoxin. Free Radic Biol Med.

[44] Wang J, Zhou JY, Wu GS (2007). ERK-dependent MKP-1-mediated cisplatin resistance in human ovarian cancer cells. Cancer Res.

